# Timely initiation of HIV antiretroviral therapy in Haiti 2004–2018: a retrospective cohort study

**DOI:** 10.26633/RPSP.2021.139

**Published:** 2021-11-19

**Authors:** Nancy Puttkammer, Canada Parrish, Yrvel Desir, Nathaelf Hyppolite, Nadjy Joseph, Lara Hall, Jean Guy Honoré, Ermane Robin, Georges Perrin, Kesner François

**Affiliations:** 1 University of Washington Washington United States of America University of Washington, Washington, United States of America; 2 National Association of State and Territorial AIDS Directors Port-au-Prince Haiti National Association of State and Territorial AIDS Directors, Port-au-Prince, Haiti; 3 Centre Haitien pour le Renforcement du Système de Santé Port-au-Prince Haiti Centre Haitien pour le Renforcement du Système de Santé, Port-au-Prince, Haiti; 4 United States Centers for Disease Control and Prevention Port-au-Prince Haiti United States Centers for Disease Control and Prevention, Port-au-Prince, Haiti; 5 Ministère de Santé Publique et de la Population Port-au-Prince Haiti Ministère de Santé Publique et de la Population, Port-au-Prince, Haiti

**Keywords:** HIV, antiretroviral therapy, highly active, implementation science, Haiti, VIH, terapia antirretroviral altamente activa, ciencia de la implementación, Haití, HIV, terapia antirretroviral de alta atividade, ciência da implementação, Haiti

## Abstract

**Objective.:**

To describe trends in timing of ART initiation for newly diagnosed people living with HIV before and after Haiti adopted its Test and Start policy for universal HIV antiretroviral therapy (ART) in July 2016, and to explore predictors of timely ART initiation for both newly and previously diagnosed people living with HIV following Test and Start adoption.

**Methods.:**

This retrospective cohort study explored timing of ART initiation among 147 900 patients diagnosed with HIV at 94 ART clinics in 2004–2018 using secondary electronic medical record data. The study used survival analysis methods to assess time trends and risk factors for ART initiation.

**Results.:**

Timely uptake of ART expanded with Test and Start, such that same-day ART initiation rates increased from 3.7% to 45.0%. However, only 11.0% of previously diagnosed patients initiated ART after Test and Start. In adjusted analyses among newly diagnosed people living with HIV, factors negatively associated with timely ART initiation included being a pediatric patient aged 0–14 years (HR = 0.23, *p* < 0.001), being male (HR = 0.92, *p* = 0.03), being 50+ years (HR = 0.87, *p* = 0.03), being underweight (HR = 0.79, *p* < 0.001), and having WHO stage 3 (HR = 0.73, *p* < 0.001) or stage 4 disease (HR = 0.49, *p* < 0.001). Variation in timely ART initiation by geographic department and health facility was observed.

**Conclusions.:**

Haiti has made substantial progress in scaling up Test and Start, but further work is needed to enroll previously diagnosed patients and to ensure rapid ART in key patient subgroups. Further research is needed on facility and geographic factors and on strategies for improving timely ART initiation among vulnerable subgroups.

## RESUMEN

**Objetivo.:**

Describir las tendencias en cuanto al momento de iniciar el tratamiento antirretroviral (TAR) de personas con infección por el VIH recién diagnosticadas antes y después de julio del 2016, cuando Haití adoptó la política de prueba e inicio del tratamiento con el TAR universal contra el VIH, y explorar los factores predictivos del inicio oportuno del TAR en personas con infección por el VIH recién diagnosticada y diagnosticada con anterioridad después de la adopción de esta política.

**Métodos.:**

En este estudio de cohortes retrospectivo se exploró el momento en que se inició el TAR en 147 900 pacientes con diagnóstico de infección por el VIH en 94 consultorios que administran TAR del 2004 al 2018 mediante datos secundarios de expedientes médicos electrónicos. El estudio empleó métodos de análisis de supervivencia para evaluar las tendencias temporales y los factores de riesgo del inicio del TAR.

**Resultados.:**

La observancia oportuna del TAR se amplió con la política de prueba e inicio del tratamiento, de tal manera que el inicio del TAR en el mismo día aumentó de 3,7 % a 45,0 %. Sin embargo, solo 11,0 % de los pacientes anteriormente diagnosticados iniciaron el TAR tras la adopción de la política. En los análisis ajustados con personas con infección por el VIH recién diagnosticadas, los factores asociados negativamente con el inicio oportuno del TAR comprendían ser un paciente pediátrico entre 0 y 14 años de edad (HR = 0,23, p < 0,001), ser varón (HR = 0,92, p = 0,03), tener más de 50 años (HR = 0,87, p = 0,03), tener un peso bajo (HR = 0,79,p < 0.001) y estar en el estadio 3 (de HR = 0,73, p < 0,001) o en estadio 4 (HR = 0,49, p < 0,001) de la enfermedad según la OMS. Se consideró la variación en el inicio oportuno del TAR según departamento geográfico y establecimiento de salud.

**Conclusiones.:**

Haití ha logrado avances considerables en la ampliación a mayor escala de la política de prueba e inicio del tratamiento, pero es necesario seguir trabajando para registrar a los pacientes diagnosticados con anterioridad y para asegurar el inicio rápido del TAR en los subgrupos de pacientes clave. Es preciso llevar a cabo investigaciones adicionales sobre los factores geográficos y los relacionados con los establecimientos y sobre las estrategias para mejorar el inicio oportuno del TAR en los subgrupos vulnerables.

## RESUMO

**Objetivo.:**

Descrever as tendências para o momento do início da terapia antirretroviral (TAR) em pessoas recém diagnosticadas vivendo com HIV antes de e após o Haiti adotar a política Testar e Tratar com a TAR universal para HIV, em julho de 2016, e analisar os preditores do início precoce da TAR em pessoas recém ou previamente diagnosticadas que vivem com HIV após a adoção da política Testar e Tratar.

**Métodos.:**

Este estudo de coorte retrospectivo analisou o momento do início da TAR de 147 900 pacientes diagnosticados com HIV em 94 ambulatórios de TAR entre 2004 e 2018, usando dados de registros médicos eletrônicos secundários. O estudo usou métodos de análise de sobrevivência para avaliar as tendências temporais e os fatores de risco para o início da TAR.

**Resultados.:**

A adoção precoce da TAR foi ampliada com a política Testar e Tratar de tal maneira que as taxas do início da TAR no mesmo dia do diagnóstico aumentaram de 3,7% para 45%. Porém, somente 11% dos pacientes previamente diagnosticados iniciaram a TAR após a política Testar e Tratar. Nas análises ajustadas entre as pessoas recém diagnosticadas vivendo com HIV, os fatores negativamente associados ao início precoce da TAR incluíram: ser paciente pediátrico de 0 a 14 anos de idade (HR = 0,23, *p* < 0,001), ser do sexo masculino (HR = 0,92, *p* = 0,03), ter 50 anos de idade ou mais (HR = 0,87, *p* = 0,03), ter peso inferior ao normal (HR = 0,79, *p* < 0.001) e estar na fase 3 da OMS (HR = 0,73, *p* < 0,001) ou fase 4 da doença (HR = 0,49, *p* < 0,001). Foi observada variação no início precoce da TAR por região geográfica e instituição de saúde.

**Conclusões.:**

O Haiti obteve avanços substanciais na ampliação da política Testar e Tratar, mas é necessário mais trabalho para inscrever pacientes previamente diagnosticados e para assegurar a TAR rápida em subgrupos-chave de pacientes. Mais pesquisas são necessárias sobre fatores geográficos e de instituições de saúde e sobre estratégias para a melhoria do início precoce da TAR entre subgrupos vulneráveis.

Haiti, a nation of 10.7 million people, has the highest burden of HIV in the Caribbean region, with an estimated 153 083 persons living with HIV (1). Among persons aged 15–49 years, HIV prevalence is 2.7% (2) and HIV/AIDS is responsible for 15.6% of deaths (3). Haiti’s Ministry of Health (MSPP) has led the expansion of HIV care and treatment programs toward the goal of achieving HIV epidemic control by 2030. As of 2018, 74.8% of people living with HIV (PLWH) in Haiti had received a diagnosis, 79.9% of diagnosed PLWH had initiated ART, and 75.9% of ART patients with an HIV viral load test were virally suppressed (1). Out of a total of 1 007 health facilities, 162 hospitals provide ART services (4).

Since 2004, Haiti’s efforts to expand ART access have aligned with World Health Organization (WHO) guidance for HIV patient management. Haiti’s January 2004 ART guidelines recommended treatment for patients with CD4 <200 cells/µL or WHO stage 4 disease. ART eligibility was expanded to those with CD4 <350 cells/µL in November 2007, and further expanded in March 2013 to those with CD4 <500 cells/µL, to all pregnant and lactating women regardless of CD4 count, and to all adults over age 50. In January 2015, ART eligibility was expanded to all those aged <5 years as well as those aged 5–14 years with WHO stage 3 or 4 disease. Finally, in July 2016, the MSPP extended universal ART to all PLWH regardless of level of HIV disease progression, an approach known in Haiti as Test and Start, or T&S. The specific T&S guidelines recommended that patients without evidence of opportunistic infections should initiate ART within seven days, or on the same day of HIV diagnosis with demonstrated ART readiness, and that symptomatic patients should initiate ART within 2–8 weeks in conjunction with clinical management of opportunistic infections (5).

Haiti’s national implementation of T&S has not previously been described. This study sought to describe trends in timing of ART initiation for newly diagnosed PLWH before and after the T&S policy change and explore predictors of timely ART initiation for both newly and previously diagnosed PLWH following T&S adoption.

## MATERIALS AND METHODS

### Study setting

This observational study examined a retrospective cohort of patients with new HIV diagnoses from January 2004 to March 2018, before and after implementation of T&S. The data covered 94 health facilities which used the iSanté electronic medical record (EMR) system and regularly reported data to a central repository at the MSPP. Sites with no EMR use after 2017 or with excessive lag in EMR data entry (defined as <80% of forms entered within 30 days of patient encounter) were excluded from the study.

### Data sources

We obtained data from iSanté and the Suivi Actif Longitudinal du VIH en Haiti data system (SALVH). iSanté is the largest EMR system in Haiti and includes approximately 70% of all ART patients (6–8). The two other EMRs widely used in the national HIV program include the Haitian Study Group for Kaposi’s Sarcoma and Opportunistic Infections (GHESKIO) and Partners in Health/Zanmi Lasanté (PIH/ZL) systems. SALVH, a comprehensive, national, longitudinal HIV case surveillance database (9, 10), receives automated name-based HIV case reports from the iSanté, GHESKIO, and PIH/ZL EMRs, as well as from all HIV testing sites in Haiti. SALVH uses this information to establish a comprehensive, de-duplicated registry of PLWH covering all persons diagnosed with HIV in Haiti. We used numeric identifiers to match the list of patients from iSanté with unique identifiers from SALVH in order to determine the date and site of first HIV-positive test and the location of the patient’s residence at the time of HIV diagnosis. We used iSanté’s longitudinal clinical data to identify patient and facility factors associated with rapid ART initiation.

### Patient population

We included all patients who registered for HIV care and treatment at an iSanté EMR site. Once diagnosed with HIV, patients are typically immediately linked to ongoing HIV care and treatment through the site’s HIV clinic, where registration and clinical intake visits are completed. Patients with new diagnoses who left a site and never completed registration within the HIV clinic were excluded from our study, as their data were not captured in iSanté. After matching iSanté patient records with the SALVH unique identifier, we identified 17 600 duplicate iSanté records (10.0% of iSanté records from study sites). Using the de-duplicated records, we identified the first-ever HIV diagnosis date for each unique patient. We excluded those who received a diagnosis at a site outside of the iSanté network, as they may have also started ART outside the network, those with missing HIV diagnosis data, those with an HIV diagnosis date after the ART start date, and those with a diagnosis date outside our study period (January 2004–March 2018).

### Measures

#### Timing of ART initiation.

Timing of ART initiation was the number of days between the first-ever HIV diagnosis date recorded in SALVH and the ART start date recorded in iSanté. We considered this as a time-to-event outcome and analyzed timing of ART initiation by year of HIV diagnosis.

#### Covariates.

To explore patient-level factors associated with timing of ART initiation, we examined several types of patient characteristics. Sociodemographic characteristics included sex (women were grouped into pregnant/lactating and non-pregnant/non-lactating categories), age at HIV diagnosis, marital status (married/cohabiting, widowed/divorced, single, or missing), Department of residence, and diagnosis in the same Department as residence. Among female patients, we determined pregnancy and lactation status at time of HIV diagnosis and ART initiation, using data from clinical assessments, obstetrical consultations, and labor and delivery records, as described elsewhere (8). Clinical characteristics included WHO stage of disease progression and body mass index, measured at the clinical intake visit or other visit within 90 days of HIV diagnosis.

Facility-level variables in our analysis were based on the health facility where HIV diagnosis occurred and included Department, category (hospital, health center with inpatient beds, health center without inpatient beds or dispensary, or missing), and ownership (public, private, mixed, or missing).

### Analysis methods

#### Timing of ART initiation and evolution over calendar time.

We determined the frequency of demographic and clinical characteristics for all patients diagnosed with HIV before and after T&S adoption. We characterized the proportion of patients with same-day ART before and after T&S adoption. To explore trends in ART initiation rates with expansion of ART eligibility criteria, we used Kaplan-Meier time-to-event analysis to estimate ART initiation rates within the first 12 months following HIV diagnosis, stratified by year of HIV diagnosis. In the time-to-event analyses, data were administratively censored as of 31 March 2018 or the date when the site-specific data were current within the central iSanté database, whichever came first.

To assess the effects of the T&S policy on ART initiation among patients who may have become newly eligible for ART under the T&S policy, we investigated patients who received an HIV diagnosis prior to 1 July 2016 but who had not started ART before this date. We used Kaplan-Meier time-to-event analysis to estimate ART initiation rates after 1 July 2016 among previously diagnosed patients, stratified by year of HIV diagnosis.

#### Patient and facility characteristics associated with timely ART initiation.

Our analysis of factors associated with timely ART initiation used a time-to-event (survival analysis) framework and considered two subgroups: 1) patients who were newly diagnosed with HIV on or after 1 July 2016 (*n* = 18 970 patients from 93 health facilities; one facility with no new HIV diagnoses after 1 July 2016 was dropped from this portion of the analysis); and 2) patients who were previously diagnosed with HIV but who had not started ART on or before 1 July 2016 (*n* = 15 024 patients from 92 health facilities; two facilities with no HIV diagnoses before 1 July 2016 were dropped from this portion of the analysis). We considered the ART initiation date as the failure date in the survival analysis framework and used health facility-specific administrative censoring dates. We used the Cox proportional hazards regression method to identify factors associated with hazard for ART initiation (11). To identify the variables to include in a multivariable Cox model, we first ran univariate Cox models and selected for inclusion all covariates with statistically significant associations with ART initiation risk at the *p* < 0.05 level. To address the multilevel nature of our data, whereby observations among patients seen at the same facility were assumed to be correlated, we used a shared frailty effect for health facility within the Cox models (analogous to a random effect for health facility in a hierarchical model) (12). Finally, we assessed for violations of the proportional hazards assumption, using post-estimation log-log plots and Schoenfield residuals (11). We used Stata 15.1 (StataCorp, College Station, TX) for the analysis.

#### Ethical review

The study received scientific and ethical review and approval from the Haiti National Committee on Bioethics and was exempted from human subjects review by University of Washington. The protocol was also reviewed in accordance with U.S. Centers for Disease Control and Prevention (CDC) human research protection procedures and was determined to be research, but CDC investigators did not interact with human subjects or have access to identifiable data or specimens for research purposes.

## RESULTS

### Patient characteristics

**FIGURE 1.**
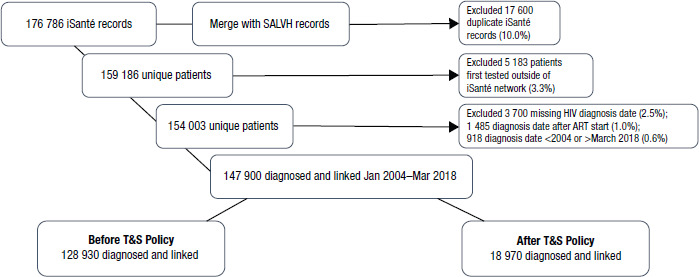
Retrospective cohort of patients newly diagnosed with HIV at 94 iSanté health facilities in Haiti (January 2004–March 2018)

***Note*:** The Suivi Actif Longitudinal du VIH en Haiti data system (SALVH) uses a combined automated and manual process to link duplicate records for all HIV case reports and HIV patient records in Haiti, as described elsewhere (10). The automated process uses deterministic matches using first name and surname, birth month and year, sex and mother’s first name, the first four letters of the patient’s first name, the reporting clinic, and the birthplace. Records matching exactly in all of these fields are automatically assigned to the same patient, missing values are not permitted to match. Human adjudication occurs for cases with a similar subset of attributes flagged through the automated process.

***Source*:** Figure prepared by the authors based on the study results.

Our study included 147 900 unique patients with HIV diagnoses from January 2004 to March 2018, with 128 930 cases before T&S adoption and 18 970 cases after T&S adoption (Figure 1). Patient characteristics are shown in Table 1. Overall, 61.7% were women, and 29.9% were estimated to be pregnant or postpartum at the time of diagnosis. Most patients were diagnosed at a testing site in the same Department as their residence (83.5%) and received HIV-related services at only one health facility (92.8%). Patients with HIV diagnoses during and after July 2016 were more likely to be male, to be children aged ≤15 years or adults aged ≥50 years, and to have WHO stage 1 or 2 disease compared to those with HIV diagnoses before this date (Table 1).

### Facility characteristics

More patients (42.7%) were managed in public health facilities than in private facilities (30.7%). Most facilities (40 facilities) were located in the West Department, followed by the North Department (12 facilities). About one-third of facilities (29 facilities) reported <5 new diagnoses per month and only 11 facilities reported >20 new diagnoses per month, on average.

**TABLE 1.** Characteristics of patients diagnosed with HIV in Haiti, January 2004–March 2018

		HIV diagnosis Jan 2004–Jun 2016 (*n* = 128 930)		HIV diagnosis Jul 2016–Mar 2018 (*n* = 18 970)		Overall HIV diagnoses (*N* = 147 900)	
	Characteristic	*n*	%	*n*	%	*n*	%
**Year of HIV diagnosis**	2004–2012	81 949	63.6			81 949	55.4
	2013	14 524	11.3			14 524	9.8
	2014	13 201	10.2			13 201	8.9
	2015	13 168	10.2			13 168	8.9
	Jan–Jun 2016	6 088	4.7			6 088	4.1
	Jul–Dec 2016			5 936	31.3	5 936	4.0
	2017			11 731	61.8	11 731	7.9
	Jan–Mar 2018			1 303	6.9	1 303	0.9
**Sex**	Female	80 078	62.1	11 197	59.0	91 275	61.7
	Pregnant/ postpartum women	23 711	29.6	3 571	31.9	27 282	29.9
	Male	48 654	37.7	7 640	40.3	56 294	38.1
	Unknown	198	0.2	133	0.7	331	0.2
**Age at HIV diagnosis, years**	0–14	13 346	10.4	2 583	13.6	15 929	10.8
	15–24	18 302	14.2	2 572	13.6	20 874	14.1
	25–34	39 822	30.9	5 483	28.9	45 305	30.6
	35–49	40 044	31.1	5 451	28.7	45 495	30.8
	≥50	14 325	11.1	2 360	12.4	16 685	11.3
	Unknown	3 091	2.4	521	2.7	3 612	2.4
**Marital status**	Married/cohabiting	60 851	47.2	9 153	48.2	70 004	47.3
	Widowed/divorced	15 437	12.0	1 928	10.2	17 365	11.7
	Single	22 450	17.4	3 746	19.7	26 196	17.7
	Unknown	30 192	23.4	4 143	21.8	34 335	23.2
**Department of residence at HIV diagnosis**	West	42 963	33.3	5 684	30.0	48 647	32.9
	North	22 291	17.3	2 741	14.4	25 032	16.9
	South	11 999	9.3	1 089	5.7	13 088	8.8
	Artibonite	11 036	8.6	2 209	11.6	13 245	9.0
	North-West	9 650	7.5	856	4.5	10 506	7.1
	North-East	6 367	4.9	994	5.2	7 361	5.0
	South-East	4 140	3.2	525	2.8	4 665	3.2
	Grand Anse	4 050	3.1	399	2.1	4 449	3.0
	Nippes	3 472	2.7	399	2.1	3 871	2.6
	Central	188	0.1	194	1.0	382	0.3
	Missing	12 774	9.9	3 880	20.5	16 654	11.3
**Location of diagnosis**	Same Department as residence	108 654	84.3	14 903	78.6	123 557	83.5

***Source*:** Prepared by the authors based on the study results.

### Timeliness of ART initiation for newly diagnosed ART patients before and after T&S

Over half of patients (58.6%) ever initiated ART, and this proportion was higher among patients who received a diagnosis after T&S adoption (77.0%) than among those who received a diagnosis before the policy change (55.8%). Comparing the periods before and after T&S adoption, same-day ART initiation rates increased from 3.7% to 45.0%.

**FIGURE 2.**
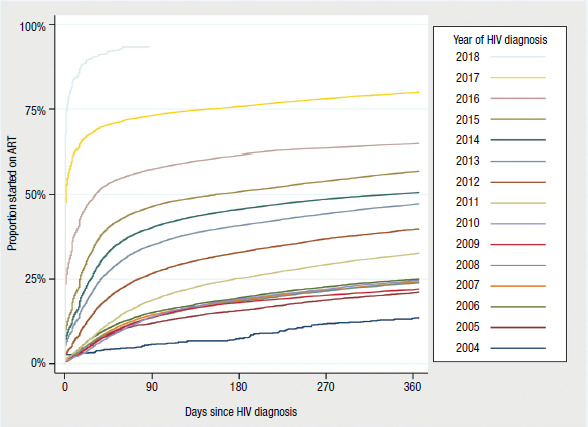
Time from HIV diagnosis to ART initiation, by year of HIV diagnosis (*N* = 147 900)

***Source***: Prepared by the authors based on the study results.

Figure 2 illustrates the trend in time to ART initiation within the first year following HIV diagnosis, by year of diagnosis based on the Kaplan-Meier method. Before the T&S policy change, the proportion of patients estimated to start ART within 30 days of diagnosis expanded from below 10% for patients diagnosed in 2004–2011, to 15.4% of patients diagnosed in 2012 (95% CI: 14.8%, 16.0%), to 35.0% diagnosed in 2015 (95% CI: 34.2%, 35.8%). After the policy change, this proportion expanded to 68.5% for those diagnosed in 2017 (95% CI: 67.7%, 69.4%) and 90.5% for those diagnosed within the first quarter of 2018 (95% CI: 88.7%, 99.2%).

### Predictors of ART initiation among newly diagnosed patients after T&S adoption

We assessed predictors of rapid ART initiation among the 18 970 patients with HIV diagnoses on or after 1 July 2016, using Cox proportional hazards regression. In bivariate Cox models, having an HIV diagnosis in the same Department as one’s residence was not associated with timely ART initiation, and no health facility characteristics were associated with timely ART initiation, so these variables were excluded from the final multivariable model. Table 2 shows hazard ratios (HR) arising from the multivariable Cox model, along with reference categories for these measures of relative risk; HRs greater than 1.0 reflect risk factors for more timely ART initiation while HRs less than 1.0 reflect risk factors for less timely ART initiation. The hazard for ART initiation increased significantly by calendar quarter following T&S adoption, after adjustment for patient characteristics. Patient characteristics associated with more timely ART initiation included being a pregnant or postpartum woman (HR = 1.12, *p* < 0.001), being 35–49 years (HR = 1.04, *p* < 0.05) or 50+ years (HR = 1.06, *p* < 0.05), being single (HR = 1.05, *p* < 0.05), and being obese at HIV diagnosis (HR = 1.10, *p* < 0.05). Patient characteristics associated with less timely ART initiation included being male (HR = 0.96, *p* = 0.04), being a pediatric patient aged 0–14 years (HR = 0.17, *p* < 0.001), and having symptoms or diagnosis consistent with WHO stage 3 or stage 4 disease (HR = 0.91, *p* < 0.01 for both). Compared with being a resident in the West Department, being a resident in the North Department was associated with more timely ART initiation (HR = 1.17, *p* < 0.01) while being a resident in the North-West Department was associated with less timely ART initiation (HR = 0.81, *p* < 0.01). The shared frailty effect in our model indicated significant variation in the baseline hazard across health facilities. Our post-estimation assessment of the proportional hazards assumption revealed violation of this assumption for the Department of residence variable; therefore, the HR estimates for this variable should be interpreted as average estimates over the time period of analysis.

**TABLE 2.** Risk factors for ART initiation following T&S adoption, among patients newly diagnosed with HIV (multivariable Cox regression model, *n* = 18 970)^*^

	Category	HR	95% CI	*p*-value
Calendar quarter (ref. = Jul–Sep 2016)	Oct-Dec 2016	1.12	(1.06, 1.19)	<0.001
	Jan-Mar 2017	1.28	(1.20, 1.35)	<0.001
	Apr-Jun 2017	1.37	(1.29, 1.45)	<0.001
	Jul-Sep 2017	1.53	(1.44, 1.63)	<0.001
	Oct-Dec 2017	1.58	(1.48, 1.69)	<0.001
	Jan-Mar 2018	1.96	(1.80, 2.15)	<0.001
**Department of residence** (ref. = West)	North	1.17	(1.06, 1.28)	<0.01
	South	1.04	(0.92, 1.17)	0.50
	Artibonite	1.01	(0.93, 1.10)	0.87
	North-West	0.81	(0.70, 0.95)	<0.01
	Nippes	1.00	(0.86, 1.15)	0.96
	South-East	0.97	(0.83, 1.14)	0.74
	North-East	1.09	(0.96, 1.25)	0.20
	Grand Anse	1.14	(0.95, 1.39)	0.17
	Central	1.05	(0.89, 1.23)	0.57
	Missing	1.01	(0.94, 1.07)	0.84
**Sex** (ref. = Female, not pregnant or postpartum)	Female pregnant or postpartum	1.12	(1.07, 1.17)	<0.001
	Male	0.96	(0.92, 1.00)	0.04
	Missing	1.46	(1.15, 1.85)	<0.01
**Age** (ref. = 25–34 years)	0-14 years	0.17	(0.15, 0.18)	<0.001
	15-24 years	0.98	(0.93, 1.03)	0.43
	35-49 years	1.04	(1.00, 1.09)	<0.05
	50+ years	1.06	(1.00, 1.12)	<0.05
	Missing	0.14	(0.11, 0.17)	<0.001
**Marital status** (ref. = Married/ cohabitating)	Widowed/divorced	0.99	(0.93, 1.05)	0.71
	Single	1.05	(1.00, 1.10)	<0.05
	Missing	1.04	(0.99, 1.09)	0.11
**WHO stage** (ref. = Stage 1)	Stage 2	1.01	(0.96, 1.05)	0.83
	Stage 3	0.91	(0.86, 0.96)	<0.01
	Stage 4	0.91	(0.85, 0.97)	<0.01
	Missing	0.50	(0.46, 0.53)	<0.001
**Body mass index** (ref. = Normal, 18.5–24.9)	Underweight, <18.5	0.97	(0.93, 1.02)	0.23
	Overweight, 25–<30	1.05	(1.00, 1.11)	0.07
	Obese, ≥30	1.10	(1.00, 1.20)	0.04
	Missing	0.76	(0.71, 0.80)	<0.001
Frailty effect^**^				<0.001

***Note*.** HR, hazard ratio; CI, confidence interval

* Analysis limited to patients with HIV diagnosis 1 July 2016–31 March 2018. Analysis of risk of ART initiation after T&S adoption contains data from 93 health facilities (one health facility was excluded because it recorded no patients diagnosed with HIV after T&S adoption).

** Likelihood ratio test of the degree of within-facility correlation of observations. This tests the null hypothesis that correlation of patient observations within facilities can be ignored. The result indicates that the baseline hazard varied across health facilities in this mixed effects survival model.

***Source*:** Prepared by the authors based on the study results.

### ART initiation after T&S among previously diagnosed patients

Among those diagnosed before the T&S policy change who had not yet initiated ART as of July 2016, ART initiation was relatively limited following T&S. Among 63 996 such patients, only 7 048 (11.0%) went on to initiate ART during the 21 months after the policy change.

### Predictors of timely ART among previously diagnosed patients after T&S adoption

Our assessment of predictors of timely ART initiation following the T&S policy change among the 15 024 patients previously diagnosed from January 2014–June 2016 revealed results somewhat different from those described above for newly diagnosed patients (Table 3, see table for reference categories). The only patient characteristic associated with more timely ART initiation included being obese at HIV diagnosis (HR = 1.26, *p* = 0.01). Patient characteristics associated with less timely ART initiation included being a pediatric patient aged 0–14 years (HR = 0.23, *p* < 0.001), being male (HR = 0.92, *p* = 0.03), being 50+ years (HR = 0.87, *p* = 0.03), being underweight (HR = 0.79, *p* < 0.001), and having symptoms or diagnosis consistent with WHO stage 3 (HR = 0.73, *p* < 0.001) or stage 4 disease (HR = 0.49, *p* < 0.001). The hazard for ART initiation increased significantly by diagnosis year, after adjustment for patient characteristics. Being a resident of the South-East Department was also associated with rapid ART initiation (HR = 1.51, *p* = 0.04). As described above, the Department of residence variable violated the proportional hazards assumption, so the estimated HR should again be interpreted as an average estimate.

## DISCUSSION

Universal ART is a critical strategy to achieve the 95–95–95 targets for HIV epidemic control. With adoption of universal treatment across many low-resource countries, observational studies using routine data sources are important for identifying successes and gaps in policy implementation, so that health outcomes can be optimized. Haiti demonstrated marked improvements in timely ART initiation from 2004 to 2018. Haiti’s acceleration in ART initiation in 2017 and 2018 matched the trajectory of implementation of universal ART in other low- and middle-income countries (LMICs) (13). Haiti’s level of same-day ART after T&S policy adoption (45.0%) compared favorably to the level found in a global survey of ART access in LMICs (38.4%) (14) but was lower than that observed in Botswana, Kenya, and Malawi (57.1%, 63.6%, and 69.5%) (15, 16). The trend toward early HIV diagnosis and rapid ART for those with WHO stage 1 and 2, following adoption of T&S in Haiti, was similar to the trend documented in Namibia following policy change (17).

Our analysis showed reduced likelihood of rapid ART among particular patient subgroups, including pediatric patients, men, and those with WHO stage 3 or 4 disease at diagnosis, for both newly and previously diagnosed PLWH. In Haiti, newly diagnosed pediatric patients aged 0–14 were 83% less likely to initiate ART at each time point following HIV diagnosis, compared to adults aged 25–34 in adjusted analyses. This concerning finding could reflect the lack of pediatric HIV services in 27% of HIV treatment sites in Haiti (requiring referral to an alternative treatment site following diagnosis) (4), delays in procedures for definitive HIV diagnosis in children (diagnostic protocols require dried blood spot samples to be sent to reference laboratories for PCR testing), delays in clinical assessment and determination of a treatment plan, higher rates of loss to follow-up after HIV diagnosis among pediatric patients, shortfalls or stock-outs in pediatric regimens, or other issues. Further research on care processes for pediatric HIV patients is needed to identify specific recommendations for streamlining ART initiation among this vulnerable subgroup.

Further research is needed to explain our findings of reduced likelihood of timely ART in the North-West Department for newly diagnosed PLWH and of increased likelihood of timely ART in the South-East Department for previously diagnosed PLWH. Compared to national averages, the number of hospital beds per capita in both North-West and South-East Haiti is low (52.1 for North-West vs. 39.9 for South-East vs. 69.8 per 100 000 for national average) (4), as is the income index (0.385 for North-West vs. 0.395 for South-East vs. 0.425 for national average) (18), meaning that these broad indicators of health access and socioeconomic status do not explain our findings. We lacked specific data on time and cost to travel to seek health services by Department, and our variable on residence within the same Department as HIV diagnosis was a weak proxy for accessibility of services, which was not associated with the outcome of ART initiation.

**TABLE 3.** Risk factors for ART initiation following T&S adoption, among patients previously diagnosed with HIV (multivariable Cox regression model, *n* = 15 024)^*^

	Category	HR	95% CI	*p*-value
**Year of diagnosis** (ref. = 2014)	2015	1.61	(1.48, 1.76)	<0.001
	Jan–Jun 2016	2.99	(2.73, 3.27)	<0.001
**Department of residence** (ref. = West)	North	1.25	(0.97, 1.60)	0.09
	South	1.27	(0.98, 1.64)	0.07
	Artibonite	1.16	(0.90, 1.49)	0.25
	North-West	1.33	(0.93, 1.89)	0.12
	Nippes	1.28	(0.91, 1.81)	0.16
	South-East	1.51	(1.03, 2.22)	0.04
	North-East	1.26	(0.93, 1.73)	0.14
	Grand Anse	1.51	(0.98, 2.33)	0.06
	Central	1.39	(0.86, 2.25)	0.18
	Missing	1.05	(0.91, 1.23)	0.49
**Sex** (ref. = Female, not pregnant or postpartum)	Female pregnant or postpartum	0.99	(0.90, 1.10)	0.89
	Male	0.92	(0.85, 0.99)	0.03
	Missing	2.25	(1.63, 3.12)	<0.001
**Age** (ref. = 25–34 years)	0–14 years	0.23	(0.19, 0.28)	<0.001
	15–24 years	1.01	(0.92, 1.12)	0.79
	35–49 years	1.07	(0.98, 1.16)	0.13
	50+ years	0.87	(0.77, 0.99)	0.03
	Missing	0.26	(0.19, 0.35)	<0.001
**Marital status** (ref. = Married/ cohabitating)	Widowed/divorced	1.03	(0.92, 1.16)	0.61
	Single	1.08	(0.98, 1.18)	0.12
	Missing	0.86	(0.78, 0.95)	<0.01
**WHO stage** (ref. = Stage 1)	Stage 2	0.91	(0.83, 1.00)	0.06
	Stage 3	0.73	(0.64, 0.83)	<0.001
	Stage 4	0.49	(0.42, 0.56)	<0.001
	Missing	1.08	(0.98, 1.20)	0.13
**Body mass index** (ref. = Normal, 18.5–24.9)	Underweight, <18.5	0.79	(0.71, 0.88)	<0.001
	Overweight, 25–<30	1.11	(0.99, 1.25)	0.08
	Obese, ≥30	1.26	(1.05, 1.50)	0.01
	Missing	0.62	(0.56, 0.68)	<0.001
Frailty effect^**^				<0.001

***Note*:** HR, hazard ratio; CI, confidence interval.

* Analysis limited to patients with HIV diagnosis January 2014–June 2016, who had not started ART by 1 July 2016. Analysis of risk of ART initiation after T&S adoption contains data from 92 health facilities (two health facilities were excluded because they recorded no patients diagnosed with HIV prior to T&S adoption).

** Likelihood ratio test of the degree of within-facility correlation of observations. This tests the null hypothesis that correlation of patient observations within facilities can be ignored. The result indicates that the baseline hazard varied across health facilities in this mixed effects survival model.

***Source*:** Prepared by the authors based on the study results.

Our analysis revealed significant variation in timely ART initiation at the health facility level. To implement T&S, multiple changes were needed at the facility level, including training health workers on the new clinical guidelines, changing processes for registering and evaluating patients for ART readiness, and disseminating new messages to patients about who should benefit from treatment and when. Many factors come into play when institutionalizing changes within health care delivery systems, such as leadership, change readiness, and health worker self-efficacy to execute new protocols (19–21); however, our data on facility attributes were limited to broad factors like facility type and ownership type, which failed to explain the variable dynamics of T&S implementation across institutions in Haiti.

The T&S policy change had limited success in ensuring ART uptake among patients with earlier diagnoses who had not yet started ART as of the T&S policy change. Under T&S, health workers attempted to recontact patients living with HIV who had not previously been eligible for ART and enroll them. However, the share of patients with previous diagnoses who went on to start ART in the 21 months after the policy change was quite small.

Key strengths of this study were the large national coverage of our data sources, reflecting approximately 70% of all patients with HIV diagnoses in Haiti, and our ability to link patient records within the iSanté data system to HIV case report data in the SALVH data system to establish valid dates for first-ever HIV diagnosis and for first ART initiation.

Our study has several limitations. First, data for WHO stage and BMI at linkage to care were missing for nearly one-third of patients. Those with missing data had a reduced likelihood of ART initiation, but this is not easily interpretable and could reflect reverse causation. The direction of bias in our estimates for WHO stage and BMI levels due to using a missing indicator category is not known (22). We also lacked data on patient-level characteristics such as education, level of HIV-related knowledge, travel distance to the clinic, mental health status, stigma, and presence of social support for treatment initiation. Any of these factors could have influenced likelihood of ART initiation. Second, we had limited data on policy implementation processes across health facilities, which prevented us from explaining the variation in ART initiation across health facilities. Third, we lacked data on patient mortality following HIV diagnosis. Among patients previously diagnosed with HIV but not started on ART at the time of our analysis, we could not distinguish those who died and those who did not start treatment for other reasons.

Our study points to the need for interventions that promote rapid ART initiation among pediatric patients, men, and those with more advanced HIV disease at time of diagnosis. This is critical given the risk of pre-ART loss to follow-up when patients do not immediately start treatment (23). Our finding of low ART initiation among previously diagnosed PLWH indicates a need for enhanced community-based outreach and tracking of patients who remain unlinked to care, as well as general community health education about the benefits of initiating HIV treatment even when feeling healthy.

### Conclusion

Our assessment of T&S implementation in Haiti identified notable progress in scaling up ART initiation among patients with new HIV diagnoses, but less progress in ART initiation among previously diagnosed patients. We also noted the important need for improvements in timely ART for pediatric patients, for men, and for patients with symptomatic disease. Strategies are needed to increase access to rapid ART among these key patient subgroups, and to ensure consistent implementation of T&S clinical guidelines across health facilities and geographic areas.

## Disclaimer.

Authors hold sole responsibility for the views expressed in the manuscript, which may not necessarily reflect the opinion or policy of the *RPSP/PAJPH* and/or those of the Pan American Health Organization. The findings and conclusions in this manuscript are those of the authors and do not necessarily represent the official position of the U.S. Centers for Disease Control and Prevention or other funding agencies.
